# *Pena, Miedo y la Desidia*: understanding US Latina farmworkers’ perspectives on breast cancer and prevention services

**DOI:** 10.1093/heapro/daag116

**Published:** 2026-08-24

**Authors:** Benjamin Aceves, Pamela Padilla, David Preciado, Ofelia Cruz, Nicolas Lopez-Galvez

**Affiliations:** School of Public Health, San Diego State University, 5500 Campanile Dr, San Diego, CA 92182, United States; School of Public Health, San Diego State University, 5500 Campanile Dr, San Diego, CA 92182, United States; Division of Research, San Diego State University Research Foundation, 5250 Campanile Drive, San Diego, CA 92182, United States; Imperial Valley Chapter, Lideres Campesinas, 319 Lambert St, Oxnard, CA 93036, United States; School of Public Health, San Diego State University, 5500 Campanile Dr, San Diego, CA 92182, United States

**Keywords:** breast cancer screening, health disparities, farmworkers, Latina or Hispanic, rural health, women’s health

## Abstract

Through a community-based participatory approach, this study leveraged semistructured interviews to qualitatively assess Latina Farmworkers’ perspectives on breast cancer and related-prevention services, specifically their knowledge and attitudes of breast cancer risk, as well as barriers and facilitators to breast cancer screening. *Promotoras* and research team members conducted 45-minute semistructured interviews in Imperial Valley (IV) from January 2024 to January 2025. A total of (*n* = 25) participants were interviewed—all identifying as female and Latina/Hispanic/Chicana. The majority of participants were aged 40–61 years (80%), and 44% reported being diagnosed with a chronic condition. Interviews were audio-recorded and transcribed verbatim, and a thematic analysis approach was utilized to construct qualitative themes. Study findings yielded four overarching themes and two subthemes: (i) Lack of transparency of chemical use and frequent pesticide exposure in the workplace creates ongoing concern for breast cancer-related risks, (i.1) Employers’ lack of transparency regarding chemical use, negligence of employees’ health, and the absence of workplace safety education place Latina farmworkers at risk, (ii) Chest injuries sustained in the workplace are perceived as critical breast cancer-related risks and heighten apprehension about women's long-term health effects, (iii) Emotional and psychological stressors discourage mammogram screenings and delay preventative health care appointments, (iii.1) Cultural dominance suppresses access to breast cancer preventative care and influences women’s overall quality of life, and (iv) Liaison between gynecologist and mammogram screenings encourages women’s preventative health practices. Interacting themes revealed barriers related to agricultural workplace exposures that are perceived as critical breast cancer-related risks, as well as emotional, psychological, and cultural stressors that frequently discourage mammogram screenings. Findings also underscored a notable liaison between gynecologists and mammogram screenings that promote women’s preventive health care practices. IV an understudied region, and findings from this study uniquely highlight the lived experiences of Latina farmworkers in this region, revealing how individual, community, and structural-level barriers not only prevent them from seeking and accessing breast cancer preventative care resources and screening services but also place them at greater risk for developing breast cancer, directly addressing the research gap existing in this area. It is imperative that culturally tailored programs and interventions, focused on education, social support, and community-clinical care coordination, are designed to overcome multilevel barriers to breast cancer care faced in this region.

## Graphical Abstract

**Graphical Abstract:**
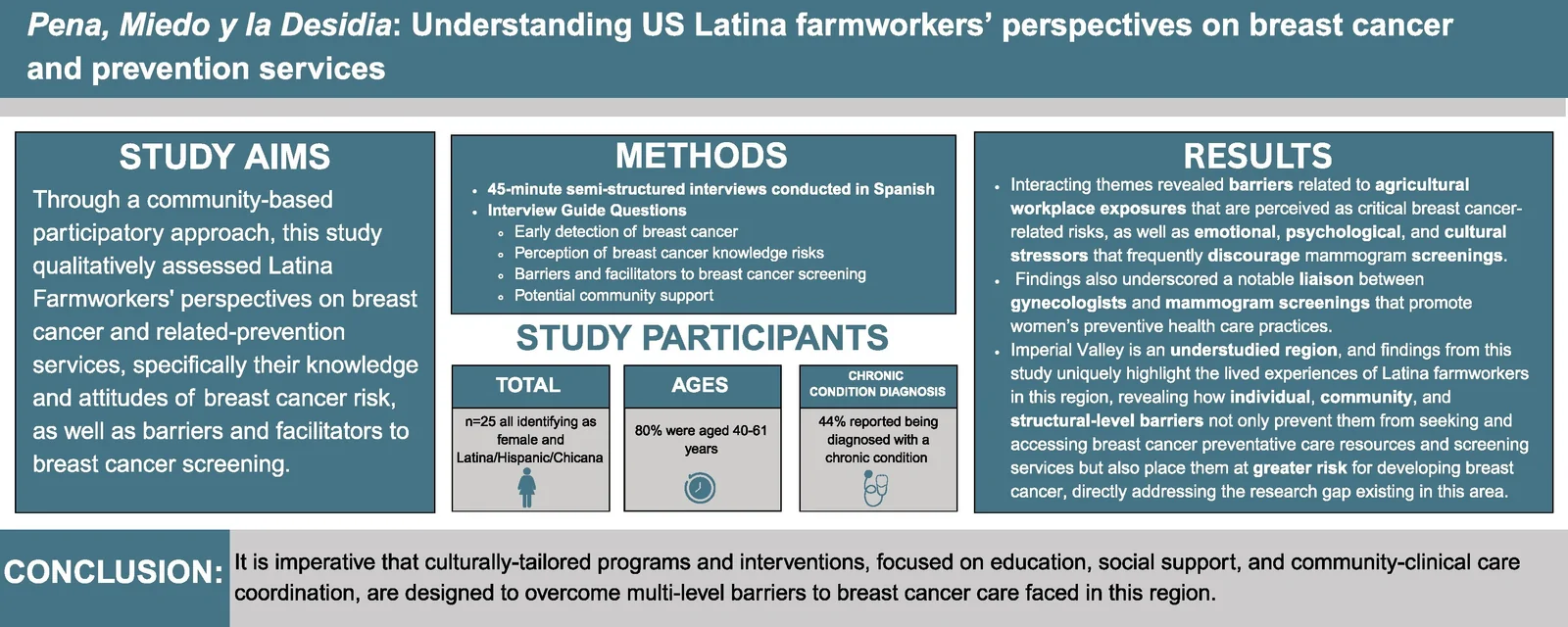

Contribution to Health Promotion • Adverse occupational experiences influence breast cancer prevention practices and related-health seeking behaviors among Latina farmworkers

• Emotional and psychological stressors discourage and delay mammograms.

• The complex interplay between clinical- and community-level factors create significant barriers to breast cancer prevention among Latina farmworkers

## Background

Breast cancer is categorized as one of the most common types of cancers and the leading cause of death among women in the world (Serrano-Gomez *et al*. 2020, Cuthrell and Tzenios 2023, American Cancer Society 2025), accounting for 30% of new cancers found among US women yearly (American Cancer Society 2025). Although incidence rates continue to rise at slow rates (1%), the American Cancer Society estimates that by 2025, ∼316 950 invasive breast cancer cases will be diagnosed among US women, followed by 42 170 breast cancer-related deaths (American Cancer Society 2025). This is particularly concerning for US migrant and rural farmworker populations, one of the most underserved populations in the nation, who not only disproportionately experience an array of socioeconomic disparities and physiological and environmental exposures but also elevated risks of breast cancer and critical barriers to breast cancer screenings (Coughlin and Wilson 2002, Schlehofer and Brown-Reid 2015, Scarinci *et al*. 2016, Furgurson *et al*. 2019, Pariser *et al*. 2022).

Breast cancer screening behaviors can be further explained by social determinants of health (SDoH) (Serrano-Gomez *et al*. 2020, Jhumkhawala *et al*. 2024). SDoH are widely defined as the conditions in which individuals grow, live, work, and age, including their access to economic means, education, and the built environment, and are frequently associated with diverse health disparities (World Health Organization 2025). Previous research has highlighted the burden US Latina women face related to breast cancer-related mortality, and how they are more likely to experience delayed breast cancer diagnosis, are less likely to receive recommended treatment regimens, are significantly underrepresented in cancer research, and experience heightened risks of breast cancer-related mortality despite their lower incidence rates (Serrano-Gomez *et al*. 2020). US Latina women are also less likely to receive breast cancer screenings compared to non-Hispanic White women (Purc-Stephenson and Gorey 2008, Serrano-Gomez *et al*. 2020). While previous research underscores breast cancer screening barriers related to cost, insurance, culture, cancer knowledge, and time constraints (Schlehofer and Brown-Reid 2015, Scarinci *et al*. 2016, Furgurson *et al*. 2019, Pariser *et al*. 2022), scarce literature uplifts US Latina farmworking women’s perspectives on the barriers and facilitators to breast cancer screenings (Schlehofer and Brown-Reid 2015). Latina farmworkers face an increased risk of breast cancer and barriers to care, due to a number of adverse SDoH rooted in their documentation status, poverty level pay, and low occupational standards for the profession and environmental exposure due to the hazardous pesticides (Schlehofer and Brown-Reid 2015, Scarinci *et al*. 2016, Furgurson *et al*. 2019, Pariser *et al*. 2022). Previous breast cancer interventions have addressed some of these issues surrounding knowledge, attitudes, and access among Latina women through leveraging community-based approaches, which were found to be effective (Oliver-Vázquez *et al*. 2002, Larkey 2006, Bittencourt and Scarinci 2019, Keith *et al*. 2019, Savas *et al*. 2021). However, more evidence is needed surrounding the risk of US Latina farmworkers given existing health disparities and barriers to health care.

Located in the southeastern corner of California, the Imperial Valley (IV) is home to a Spanish-preferring (73.1%), Hispanic/Latino (85.4%), and a large farmworker population (9.8%) compared with the overall California population (2.1%) (Imperial County Health Improvement Partnership 2024). A recent Community Health Assessment conducted in 2024 among IV residents reports that Hispanic/Latino-identifying residents need improvements related to healthcare provider access to improve their overall health (46%) (Imperial County Health Improvement Partnership 2024). Additionally, Hispanic/Latino-identifying residents reported not getting regular health screenings (26%) and not having adequate specialty medical care in the IV region (27%) (Imperial County Health Improvement Partnership 2024). Considering IV hosts a large Latino and agricultural population, it is critical to explore the unique perspectives on breast cancer screenings in this region to further understand and identify compelling health priorities.

*Promotoras* are notable, trustworthy figures in studies serving Hispanic/Latino populations, known for their strong leadership skills, communication expertise, and personal characteristics, critical for the delivery of culturally and linguistically appropriate services and addressing health disparities (Koskan *et al*. 2013, Schwingel *et al*. 2017). Although previous studies have demonstrated successful *promotora* engagement with farmworking communities to address health-related disparities (Ingram *et al*. 2007), no studies have leveraged promotora support to document farmworker women's perspectives on breast cancer risk and screening in IV.

Using a community-based participatory research approach, the present study aims to qualitatively assess IV farmworker women's knowledge and attitudes of breast cancer risk, and to illustrate barriers and facilitators to breast cancer screening.

## Methods

The Institutional Review Board at San Diego State University reviewed and approved all study protocols, designs, and materials. Written informed consent was obtained from all participants. All procedures in this research were conducted in accordance with the ethical standards of the Helsinki declaration. We partnered with the IV chapter of the statewide network *Lideres Campesinas* (Lideres Campesinas en California 2024) to lead recruitment efforts and conduct 25 semistructured interviews among Latina farmworkers in the IV. *Lideres Campesinas* is a state-wide nonprofit that advocates and supports agricultural communities across California (Lideres Campesinas en California 2024). *Promotoras* from *Lideres Campesinas* leveraged existing social networks in Imperial County to engage with community members and applied snowball and convenience sampling techniques to recruit study participants. *Promotoras* or community health workers (CHWs) are key members of public health teams and act as critical liaisons between community members and local resources and services (Ayala *et al*. 2010, Nelson et al. 2011; Schwingel *et al*. 2017). Within the scope of this study, *promotoras* were engaged in the entire process from the onset of the development of the interview guide and establishment of protocols for the study, to conducting the interviews, and the analysis of data by engaging in an iterative process of theme development and manuscript preparation.

Eligible participants confirmed they were (i) residents of IV, (ii) aged 40 years or older, and (iii) worked in agriculture at the time of the study. All interviews were conducted from January 2024 to January 2025 by bilingual research assistants and *Lideres Campesinas Promotoras*, utilizing a semistructured interview guide with a duration of ∼45 minutes. All interviews were conducted in Spanish, and questions from the interview guide were organized into four parts: (i) early detection of breast cancer, (ii) perception of breast cancer knowledge of risks, (iii) barriers and facilitators to breast cancer screening, and (iv) potential community support. Interviews were audio-recorded and transcribed verbatim through a professional transcription service. Participants were offered a $100 gift card for their participation in the interviews.

Two bilingual research team members (PP and DP) utilized the *Dedoose* (*Version 9.0.17*) application to conduct a content qualitative analysis across all interview transcripts (Dedoose; Hsieh and Shannon 2005). Interview transcripts were uploaded into the Dedoose software to be analyzed in their original Spanish language. Key steps involved data categorization, codebook creation, individual blind-coding of the data, and acquiring 100% code agreement by coders (Cliodhna and O'Connor 2020; Hsieh and Shannon 2005). This agreement was achieved by coders collectively reviewing all study transcripts and coded data, while verbally discussing existing discrepancies in the codes, and reaching an agreement where codes may have differed in coded transcripts. The codebook consisted of codes surrounding barriers and facilitators to breast cancer care, breast cancer knowledge, lived experiences, community needs, social support, medical history, and workplace exposures. Analysts (PP and DP) applied a thematic analysis approach by iteratively identifying patterns and nuances in the coded data and constructed themes across data sources (Guest *et al*. 2012). These themes were iteratively developed in partnership with the larger study team that included *promotoras* and study staff. Participants’ demographic data (i.e. gender, age, country of origin, and chronic disease diagnosis), transcripts, and field notes were triangulated to further contextualize participants’ perspectives on breast cancer risk and lived experiences navigating breast cancer-related care (Carter *et al*. 2014). All study team members reviewed and approved the interacting themes presented in the results section, and the quotes featured were translated into English by bilingual analysts (PP and DP) to support our findings.

## Results

### Participant demographics

All study participants identified as female (100%). The average age of interviewed participants was 53.68, and all participants identified as Latina (100%). Mexico was identified as the country of origin for most participants (92%); however, no participants identified with an indigenous group. Notably, nearly half of the interviewed participants identified as being diagnosed with a chronic disease (44%). All demographic information can be referenced in Table 1.

**Table 1:** Participant demographics (*n* = 25).

Measure	Item	Count (no.) (*n* = 25)	Percentage (%)
Gender	Female	25	100
Age	40–50	10	40
	51–61	10	40
	62–72	5	20
Country of origin	Mexico	23	92
	United States	2	8
Identify as Hispanic/Latina/Chicana	Yes	25	100
	No	0	0
Identify with an indigenous group	Yes	0	0
	No	25	100
Diagnosed with chronic disease	Yes	11	44
	No	14	56

Theme 1.0 Lack of transparency of chemical use and frequent pesticide exposure in the workplace creates ongoing concern for breast cancer-related risks.

Participants shared their perspectives on numerous factors that they strongly believe exacerbate their risk of breast cancer. When team members asked participants to identify breast cancer-related risks, they frequently recalled pesticides and chemicals from agricultural fields. Notably, feelings of uncertainty and concern were observed among participants, who demonstrated awareness of the frequent exposure to chemicals in the workplace, but were unsure of the specific chemicals sprayed and their potential health impacts.

‘On the field, there are cancer risks because of the chemicals, and we are very close to these chemicals. We are bent down and we joke around, and say we have them [chemicals] on our hands. That’s why they tell us to wash our hands- but we don’t even know what those chemicals are’ (Participant #20, Age 64).

‘It could be hereditary, but it could also be from the chemicals at work, right? There are always chemicals in the soil from the spraying, and they tell us to stay away. We are always exposed to chemicals…I have been working in the field for 37 years’ (Participant #1, Age 65).

‘We don’t even know what they spray there, but we know they spray because we see them dressed in white suits and tractors spraying [the field]’ (Participant #25, Age 58).

As a result, participants believe their constant exposure to pesticides in the field could increase their susceptibility to a variety of illnesses.

‘I think we do run the risk of developing any type of illness from these chemicals’ (Participant #14, Age 41).

Subtheme 1.1 Employers’ lack of transparency regarding chemical use, negligence of employees’ health, and the absence of workplace safety education place Latina farmworkers at risk.

Participants expressed feeling hesitant toward challenging their employers’ lack of transparency regarding the kind of chemicals they are exposed to in the field. Although their employers, also referred to as ‘stewards’ or ‘mayordomos,’ have sometimes provided training on personal protective equipment (i.e. gloves, masks), they do not disclose the type of chemicals used when spraying pesticides in the workplace.

‘…To wear masks and gloves, and sometimes they provide them for us’ (Participant #21, Age 42).

Some participants mentioned that at times, their stewards are also unaware of the kind of pesticides being utilized.

‘Sometimes they tell us one day before, and sometimes they tell us that same morning that they closed that area because there are pesticides. I have asked, but sometimes the same stewards don't even know’ (Participant #23, Age 49).

Other participants reported being threatened with employment termination and loss of future opportunities if they requested information or clarification about their workplace exposures.

‘If we begin with those topics, they will say, ‘We aren’t going to give you work anymore.’ That happened to me…they didn't want to give me a job because our boss doesn’t want that, he wants people who come to do what they're supposed to do and work, and that’s it… ‘Don’t hire her because she will complain!’ (Participant #14, Age 41).

Participants also expressed how their employers do not prioritize the longevity of their employment, resulting in the negligence and absenteeism of workplace safety education and transparency.

‘Every day, they change employees; they don’t focus on that. Some work one day or two and then don't come back, so they [stewards] are not interested in keeping us informed’ (Participant #1, Age 65).

Theme 2.0 Chest injuries sustained in the workplace are perceived as critical breast cancer-related risks and heighten apprehension about women's long-term health effects.

Participants echoed enduring a series of impactful work-related injuries and identified them as critical breast-cancer-related risks. When team members encouraged participants to share their perspectives on breast-cancer-related causes, participants recounted experiences where they endured labor-related injuries on the field, specifically chest-related injuries. Other participants shared experiences where they had critical medical procedures performed to extract breast cysts and strongly believed these cysts developed from work-related injuries sustained during their time in the field. As a result, feelings of anguish and concern were observed as participants believed that these chest-related injuries increased their risk of developing breast cancer and were concerned about the long-term impacts of these occupational exposures.

‘They throw the melons at us and hit us in our breasts. Corn is thrown at us, and the tip of the corn hits us, and it makes us want to bend down and cry. These are the kind of blows that can sometimes provoke little balls, and those little balls can be that [breast cancer]’ (Participant #1, Age 65).

‘The only risk I see is the blows. Those blows women sustain because we have soft parts. I had [cysts] when I worked in the field, and I would hit myself with the columns’ (Participant #18, Age 61).

‘For us, the work in the [field]…it has to be from the blows. Sometimes others don’t pay attention, or we’ll hit ourselves with the boxes, which can have a lot to do. Many years ago, I got a bruise, but it went away’ (Participant #24, Age 57).

Theme 3.0 ‘*Pena, Miedo, y La Desidia*’ (Fear, Shame, and Avoidance): Emotional and psychological stressors discourage mammogram screenings and delay preventative health care appointments.

Emotional and psychological factors such as fear, shame, and avoidance were perceived barriers to seeking breast cancer screenings. Participants’ interpersonal connections (i.e. family, community members) were observed as notable influencers contributing to decreased self-efficacy, oftentimes creating a negative perception and fear-based perspectives of mammograms, and discouraging annual screening practices. Additionally, participants often feared the pain associated with the procedure and the determination of their results.

‘People don’t take the exam seriously enough and make excuses, ‘Ugh, I don’t have time, I’ll go tomorrow, tomorrow I’ll go. It’s an exam’ (Participant #12, Age 56).

‘I tell you, my sister always reminds me, ‘[Breast cancer mamograms] always hurts.’ Aside, the truth is, I fear having to confront, ‘What if something comes up?’ (Participant #7, Age 44).

Another notable barrier to mammogram screening was feelings of embarrassment, vulnerability, and shame of exposing their bodies during the examination process.

‘They say being embarrassed [is a barrier to screenings]…Well, they examine your breasts. We are women. And many don’t get exams, because they are embarrassed’ (Participant #9, Age 55).

Other participants revealed how their agricultural occupation and economic need act as critical barriers to seeking breast cancer screening services. Participants are unable to take a day off for healthcare-related services, given that they will not get compensated by their employer. As a result, participants are hesitant to seek preventative health behaviors and neglect their healthcare.

‘In the field, there are many people who do not want to miss work because they need the money. For that reason, they say ‘I’ll do it another time’ or they won’t do it at all, ‘I don’t have time’ (Participant #1, Age 65).

Subtheme 3.1 ‘*Somos Mujeres*’ (We are Women): Cultural dominance suppresses access to breast cancer preventative care and influences women’s overall quality of life.

Cultural gender roles that limit women’s bodily autonomy were identified as critical barriers to accessing breast cancer preventive health care services. Participants echoed how numerous farmworking women struggle to seek women’s cancer preventative healthcare services, like mammograms and pap smears, because their husbands forbid them from being examined by another person, including medical providers.

‘If [she’s] going to get checked, they're going to do a pap smear or a mammogram, [her husband] has to be there…No, you can't go get checked, you can't. And if she goes to the doctor, she is there and he is on the side and he is holding her hand…she says that she is very afraid of answering something that is asked of her’ (Participant #11, Age 58).

Additionally, participants assured that within breast cancer preventative care, it is crucial to engage both men and women due to the husbands’ involvement with their wives’ bodily care. Various participants revealed that they are only able to schedule their mammograms when their husbands are present. Feelings of neglect and abandonment were observed as participants shared narratives where husbands left their wives with terminal cases or when a mastectomy was performed.

‘Both women and men, because many men do not let their wives go to get treatment. Or they already leave them since they are already dying, they already see that it is something bad, or else many husbands leave them too when they cut off their breasts’ (Participant #3, Age 50).

Theme 4.0: Liaison between gynecologist and mammogram screenings encourages women’s preventative health practices.

A notable liaison was observed among participants’ gynecologists and mammogram screenings, serving as facilitators toward practicing preventative health care routines. Participants collectively shared how, at the time of their routine pap smear examination, their gynecologist or medical providers would encourage them to get screened for breast cancer.

‘Each year, my gynecologist requests my mammogram screening and my pap smear’ (Participant #18, Age 61).

‘I went in for my check-up with my gynecologist, an abnormal pap smear, and because of my age, they sent me to get a mammogram’ (Participant #2, Age 46).

‘She has a gynecologist and they do her mammogram and pap smear’(Participant #10, Age 62).

Medical providers would assure them it's a routine screening, and mentioned how mammograms are encouraged at certain ages among women.

‘When I was going to turn 40, my gynecologist told me I needed to get examined. I asked if something was wrong, but she said, ‘No, it’s routine, so see how you are,’ so I felt at ease’ (Participant #5, Age 44).

At times, medical staff would schedule their mammogram appointment directly after their pap smear examination, further facilitating their navigation process and encouraging participants to seek their annual screening.

‘My gynecologist makes my appointment, ‘Hey, it’s due, I’m going to schedule you for your mammogram appointment,’ and they automatically make it for me, and I have no issues’ (Participant #19, Age 57).

## Discussion

Similar to previous research, numerous factors that act as critical individual-, community-, and policy-level barriers to breast cancer screenings were identified in this study (Buki et al. 2004; Schlehofer and Brown-Reid 2015). Our findings also confirm how Latinas are hesitant to seek annual breast cancer screening due to existing emotional triggers like embarrassment and fear (Buki et al. 2004; Schlehofer and Brown-Reid 2015, Tejeda *et al*. 2009). The embarrassment is oftentimes associated with Latino cultural norms of female modesty, where the exposure of reproductive organs (i.e. breasts) for medical examinations is deemed as immodest or inappropriate, especially if the medical provider identifies as male (Schlehofer and Brown-Reid 2015). Additionally, the concept of cultural dominance (i.e. machismo) emerged when participants expressed being restricted or discouraged by their partners from seeking women’s preventative health care screenings, as these screenings require examination of sexualized organs by medical providers (Pariser *et al*. 2022). Similar studies have also mentioned how cancer knowledge, insurance status, cost, culture, transportation, time constraints, and fear act as barriers to Latina farmworking women’s preventative health care services, but how socio-political structure factors create barriers to breast cancer screenings (Pariser *et al*. 2022).

This study uniquely revealed that Latina farmworkers face additional socio-political structural barriers to breast cancer screening, which are rooted in the economic exploitation of farmworkers as a labor force and the political infringement of workers’ rights. Latina farmworkers worried about the wages lost in taking time to receive a mammogram and not being provided ‘sick hours’, in addition to the cost of the services–even though it is covered in California by publicly available funding (California Department of Health Care Services 2026)—are faced with significant barriers to breast cancer prevention. Prohibiting workers from sick hours is clearly a violation of California Labor Laws, given the law states that regardless of their country of origin or immigration status, workers are entitled to take time off to seek medical attention (State of California Department of Industrial Relations 2026), yet our participants describe those rights being deprived from agricultural workers. The intersectional barriers of Latina farmworkers’ social needs (i.e. lack of access and economic instability) and employer noncompliance with labor laws are clearly a structural barrier to care that needs to be addressed, given it consequently lags breast cancer screening and as a result may delay prognosis—placing this population at risk for identifying breast cancer at later stages.

Participants also mentioned being exposed to physical and chemical hazards in the workplace. While there is a lack of scientific literature linking physical impact to the development of breast cancer, these perceptions persist and therefore demonstrate a gap in knowledge surrounding the breast cancer-related risks. However, in terms of chemical exposure (pesticides), which can increase risk significantly, participants describe being withheld vital information surrounding the kinds of chemicals they are exposed to in the process of farmworking. Participants are already acutely aware of the risk of developing cancer, including breast cancer, from their exposure to carcinogenic pesticides; however, they lack the knowledge on how to best protect themselves during this hazardous exposure. This is in stark contrast to current regulations by the United States Environmental Protection Agency (EPA), which requires specific information to be displayed in a central location at the workplace: (i) treatment location and description, (ii) product name with EPA registration number and active ingredient (s) of the pesticide, (iii) time and date of pesticide application, and (iv) a restricted entry interval for the pesticide. This information is required to be posted before the application of any pesticides and when agricultural employees are on-site (United States Environmental Protection Agency 2025). It is critical to note that certain problematic exemptions exist in the Agricultural Worker Protection Standard (WPS) (United States Environmental Protection Agency 2025). Clearly, based on these experiences, more regulatory efforts need to be implemented for the safety of all farmworkers. In addition, given these Latina farmworkers saw chest injuries as a primary factor for developing breast cancer, it is urgent to provide more evidence-based education to this population to support breast cancer prevention efforts.

### Strengths and limitations

Our community-based methodological approach was a significant strength of the study. Given this qualitative research was conducted in a culturally sensitive manner and was comprised of bilingual *promotora* team members with extensive experience working with farmworkers, we were able to engage in personable conversations (*platicas*) to better understand participants’ lived experiences, perspectives, and comprehend some of the issues faced by Spanish-speaking farmworkers (Nelson et al., 2011; Ojeda *et al*. 2011). Our collaboration with *promotoras* (CHWs) from *Lideres Campesi*nas, identifying as Latina women, and strong advocates for farmworking communities, created trust (*confianza*) with participants, and *promotoras* carefully considered participants’ values, beliefs, and traditions throughout their conversations with participants (Ojeda *et al*. 2011). Previous research has highlighted how Latina women feel more comfortable being interviewed by female-identifying researchers (Ojeda *et al*. 2011). The use of *promotoras* during our interview process allowed participants to feel comfortable discussing sensitive health topics and how their culture and occupation play critical roles in seeking women's preventative healthcare services (Ojeda *et al*. 2011). Limitations of the study include only being able to engage with Latina farmworkers who had the time to meet with study personnel; unfortunately, due to the demanding nature of farmwork, this may have inherently excluded certain folks with time constraints. In addition, this study was conducted in California, which politically is more supportive of farmworkers’ rights and access to healthcare; however, in the context of other states, Latina farmworkers may face additional barriers.

## Conclusion

### Recommendations and future directions

Overall, Latina farmworkers clearly face a multitude of individual-, community-, and policy-level barriers related to breast cancer prevention practices, resources, and screening. Previous interventions have attempted to address some of these issues through targeted interventions; however, several of these interventions still need to more concretely address complex clinical-community level factors that interplay to prevent access to breast cancer prevention among Latina farmworkers (Rodriguez *et al*. 2020, Liu *et al*. 2023). This was achieved through comprehensively understanding this complex phenomenon through the integration of *promotoras* throughout the research process. This also highlights a need for more comprehensive programs and interventions that provide education, social support, and community-clinical care coordination for Latina farmworkers to support them in navigating these multilevel barriers to breast cancer care.

## Data Availability

Given the sensitivity of the data, no datasets will be publicly available for this study.
